# Comparative Genomic Analysis of Two Multidrug-Resistant Clinical Isolates of ST395 Epidemic Strain of *Pseudomonas aeruginosa* Obtained 12 Years Apart

**DOI:** 10.1128/genomeA.00515-14

**Published:** 2014-05-29

**Authors:** Benoit Valot, Laurence Rohmer, Michael A. Jacobs, Samuel I. Miller, Xavier Bertrand, Didier Hocquet

**Affiliations:** a UMR 6249 CNRS Chrono-environnement, Université de Franche-Comté, Besançon, France; b Department of Microbiology, University of Washington, Seattle, Washington, USA; c Department of Genome Sciences, Immunology, Medicine, University of Washington, Seattle, Washington, USA; d Infection Control Department, University Hospital, Besançon, France; e Biological Resources Center–Ferdinand Cabanne, University Hospital, Besançon, France

## Abstract

*Pseudomonas aeruginosa* can cause large and prolonged outbreaks in hospitals. We have sequenced and annotated the genomes of two multidrug-resistant *P. aeruginosa* isolates from the same strain obtained 12 years apart from different patients. Genomic analysis provided insight on the genes acquired and lost by *P. aeruginosa* during its spread.

## GENOME ANNOUNCEMENT

*Pseudomonas aeruginosa* is a common opportunistic pathogen in humans that causes serious infections, especially in neutropenic and critically ill patients. It is intrinsically resistant to many antibiotics and can become resistant to all of the antipseudomonal agents. Clonal outbreaks of multidrug-resistant *P. aeruginosa* affecting hospitals are often described (1, 2). The University Hospital of Besançon (France) has been hit by a 12-year *P. aeruginosa* outbreak with an international high-risk clone of sequence type 395 that has affected 250 patients. The antibiotic resistance profile of the isolates varied over time (3), along with colony morphology, suggesting an adaptation process that included, but was not limited to, the antibiotic pressure. We aimed to decipher the genetic determinants that drive this evolution during its spread into the hospital setting.

The first isolate of the outbreak, DHS01, was isolated in May 1997 from the nose of a colonized patient in the surgical intensive care unit (SICU). This strain was susceptible to all β-lactams and resistant to fluoroquinolones and aminoglycosides, with the exception of amikacin. The later isolate, DHS29, was isolated in April 2008 from the urine of another SICU patient. It was resistant to all β-lactams and to fluoroquinolones while susceptible to aminoglycosides. Genomic DNA from the DHS01 and DHS29 strains was sequenced on an Illumina GAIIx platform to generate 22,539,003 and 21,692,025 reads, respectively. The reads were assembled with Velvet version 1.1 (4) into the draft genomes of isolates DHS01 (6,953,788 bp, 106 contigs, and an *N*_50_ of 159,116) and DHS29 (7,108,323 bp, 267 contigs, and an *N*_50_ of 66,667). The two genomes had similar G+C contents of 65.8%. Genome annotation was performed by the NBCI Prokaryotic Genome Annotation Pipeline (http://www.ncbi.nlm.nih.gov/genome/annotation_prok/) and predicted 6,646 genes, 4 rRNAs, and 61 tRNAs for DHS01 and 6,807 genes, 4 rRNAs, and 55 tRNAs for DHS29. Comparison of the two genomes with Mummer software (5) revealed 1,446 single nucleotide polymorphisms (SNPs) and 1,197 insertions/deletions (indels). We determined the specific regions of the two isolates using a cross-mapping of raw reads and assembled contigs using the Maximum Entropy Method (MEM) algorithm of the BWA software (6). Seventeen genes were specific to DHS01 and 136 to DHS29. A gene encoding a 2′-aminoglycoside nucleotidyltransferase was present in the aminoglycoside-resistant DHS01 and absent from the aminoglycoside-susceptible DHS29. The DHS29-specific genes were grouped into three clusters, of which two were similar to the regions *pa1247-pa1292* and *pa1381-pa1449* of the reference strain PAO1 (7), and included the coenzyme B12 biosynthesis gene cluster, the flagellum biosynthesis cluster *fli*, and the quorum-sensing-associated genes *lasR*, *rsaL*, and *lasI*. The absence of *lasR* in DHS01 was consistent with the iridescent sheen surface of its colonies on rich agar media (8). We show here that during its spread into a hospital setting, an epidemic clone of *P. aeruginosa* not only accumulates numerous SNPs and indels but also acquires or loses gene clusters.

### Nucleotide sequence accession numbers.

These whole-genome shotgun projects of *P. aeruginosa* strain DHS01 and DHS29 have been deposited at DDBJ/EMBL/GenBank under the accession numbers AYNE00000000 and AYON00000000, respectively.
